# Retraction: Ventricular tachyarrhythmia risk in paediatric/young vs. adult Brugada Syndrome patients: a territory-wide study

**DOI:** 10.3389/fcvm.2024.1532091

**Published:** 2024-11-28

**Authors:** Frontiers Editorial Office

A Retraction of the Brief Research Report Article Ventricular tachyarrhythmia risk in paediatric/young vs. adult Brugada Syndrome patients: a territory-wide study by Lee S, Wong WT, Wong ICK, Mak C, Mok NS, Liu T and Tse G (2021). Front. Cardiovasc. Med. 8:671666. doi: 10.3389/fcvm.2021.671666

Following publication, concerns were raised regarding the ethical approval for this study. An investigation was conducted by Hong Kong Hospital Authority Research Ethics Committee (REC). This committee confirmed that this study did not receive appropriate ethical approval.

An investigation was then conducted in accordance with Frontiers' policies, and it was also confirmed that, contrary to the statement in the article, the study did not receive appropriate ethical approval. Lack of appropriate ethical approval is a breach of Frontiers' guidelines and those of the Committee on Publication Ethics. As such, this article is being retracted.

The retraction was approved by the Chief Editors for Frontiers in Cardiovascular Medicine and the Chief Executive Editor of Frontiers. The authors do not approve of the retraction.

